# Carbapenemase-producing Acinetobacter baumannii in a surgical intensive care unit – take time for tracking

**DOI:** 10.1186/2047-2994-4-S1-P137

**Published:** 2015-06-16

**Authors:** T Götting, W Ebner, D Jonas, A Serr, G Häcker, M Dettenkofer

**Affiliations:** 1 University Medical Center Freiburg, Freiburg, Germany; 2 Gesundheitsverbund Landkreis Konstanz, Singen, Germany

## Introduction

Although the prevalence of carbapenemase-producing *Acinetobacter baumannii* (CP-AB) in Germany is still low an increasing number of outbreaks has been reported in recent years. In August 2014 we observed a cluster of 4 patients with CP-AB (OXA-23) on our surgical intensive care unit (SICU).

## Objectives

After excluding an environmental source we performed epidemiological analyses to gain insight into the spread of CP-AB in our university hospital.

## Methods

To characterize the CP-AB isolates molecular typing using amplified fragment length polymorphism was conducted. A genotypic comparison with strains collected in 2013 and the first half of 2014 was carried out. All new patient isolates of CP-AB were genotyped prospectively.

## Results

Molecular typing revealed that 3 out of the 4 SICU-isolates were genotypically identical. Although temporally and locally coinciding, the 4th isolate of the patient cluster did not match.This 4th strain however was identical to a strain previously identified in a patient from the neurological department in July 2014. A physical link between these two patients could not be identified. Even more surprisingly, another isolate of CB-AB from a neurosurgical patient isolated in September 2014 was found to be identical with the three isolates of the SICU cluster, despite the absence of detectable temporal or local link to the SICU.

## Conclusion

Isolates of CP-AB from six patients fell into two clusters of two and four strains, respectively.

In this case patient transfer within the hospital cannot explain these unexpected genotypical relationships between isolates from three departments. Transmission of CP-AB may have occurred in the context of consultants’ visits or when patients were temporarily moved to a diagnostic unit such as endoscopy or radiology.

In the context of clusters of CP-AB isolated from several patients, genotyping provides the opportunity to follow the movement of these highly resistant bacteria within a hospital. Surprising genotyping results should engender efforts to analyse the pathways of transmission.

## Disclosure of interest

None declared.

